# Changes in protein profiles of guinea pig sclera during development of form deprivation myopia and recovery

**Published:** 2010-10-27

**Authors:** Xiangtian Zhou, Juxiu Ye, Mark D.P. Willcox, Ruozhong Xie, Liqin Jiang, Runxia Lu, Jianzhen Shi, Yan Bai, Jia Qu

**Affiliations:** 1 School of Optometry and Ophthalmology and Eye Hospital, Wenzhou Medical College, Wenzhou, Zhejiang, China; 2 State Key Laboratory Cultivation Base and Key Laboratory of Vision Science, Ministry of Health P.R. China and Zhejiang Provincial Key Laboratory of Ophthalmology and Optometry, Wenzhou, Zhejiang, China; 3 School of Optometry and Vision Science, The University of New South Wales, Sydney, NSW, Australia; 4 Brien Holden Vision Institute, Sydney, NSW, Australia; 5 Vision Cooperative Research Centre, Sydney, NSW, Australia

## Abstract

**Purpose:**

To investigate changes in protein profiles of posterior sclera in guinea pigs during development of form deprivation myopia and recovery.

**Methods:**

Three groups of guinea pigs (developing form deprivation myopia, recovering from the myopia and normal control) were evaluated for protein profiles of the posterior sclera using two-dimensional gel electrophoresis. Protein spots with a different intensity of at least threefold among the 3 groups were further identified with mass spectrometry. Key proteins associated with ocular growth (crystallins) were examined at mRNA levels using RT–PCR.

**Results:**

Moderate myopia was induced at 7 weeks of monocular deprivation and then more gradually recovered toward the previous refractive status 4 days after re-exposure of the eye to normal visual conditions. The profile of all protein spots at the posterior sclera was similar for both the deprived and the recovery eyes but distinct between either of the 2 experimental eyes and the normal control eyes. Twenty-six and 33 protein spots were differentially expressed in the deprived and the recovery eyes, respectively, compared to the normal control eyes. In contrast, the number of proteins differentially expressed between the deprived and the recovery eyes was only 5. Among the different subtypes of crystallins, βB2-crystallin was down-regulated and βA4-crystallin was upregulated in the deprived eyes at both protein and mRNA levels compared to the normal control eyes. The trend of expression for βA3/A1-crystallin was also similar at both mRNA and protein levels for the deprived eyes. However, αA-crystallin mRNA in the recovery eyes was upregulated while αA-crystallin itself was down-regulated. A similar inconsistency in expression of βA3/A1-, βA4-, and βB2-crystallins between the protein and mRNA levels also occurred in the recovery eyes.

**Conclusions:**

Proteomic analysis provides a useful survey of the number of proteins whose levels change during form deprivation myopia and the subsequent recovery. In particular, the crystallins changed during the development of form deprivation myopia and recovery. The changes in crystallin protein levels were consistent with that in mRNA levels during the development stage of form-deprivation myopia (FDM). However, the changes of most crystallin protein levels were mismatched with mRNA levels during the recovery stage.

## Introduction

Myopia is one of the most prevalent ocular conditions affecting visual acuity, in which images of distant objects are brought to a focus in front of the retina resulting in blurred vision. In most of cases the structural cause of myopia is an excessive axial length of the eye, or more specifically the excessive vitreous length [1-6]. The excessive axial elongation of the eye, by necessity, must involve the outer coat of the eye, the sclera. The sclera is a dense, fibrous, viscoelastic connective tissue, which in addition to protecting the retina and allowing the attachment of the extraocular muscles, controls the size of the eye and the location of the retina relative to the focal plane, and consists of irregularly arranged lamellae of collagen fibrils interspersed with proteoglycans and non-collagenous glycoproteins.

The sclera is not a static container of the eye, but rather is a dynamic tissue, capable of altering its extracellular matrix composition and its biomechanical properties in response to changes in the visual environment to regulate ocular size and refraction [7]. High myopia is characterized by scleral thinning and localized ectasia of the posterior sclera. Many studies [8-11] have demonstrated scleral changes in both experimental myopic and clinical conditions [1-6]. Some genes have been found to be closely related to high myopia such as matrix metalloproteinases [12], PAX-6 (paired box gene 6) [13,14], myocilin [15], TGF-β1 (transforming growth factor, beta 1) [16], Collagen type I alpha 1 [17], Rasgrf1 (Ras protein-specific guanine nucleotide-releasing factor 1) [18], and GJD2 (gap junction delta-2 protein) [19]. Morphological changes result from physiologic changes and physiologic activities in a tissue are basically performed by relevant functional proteins. Therefore, functional proteins in the eye are directly responsible for ocular morphological changes in response to any triggering factors. Thus, investigation of the posterior sclera tissues is necessary to understand mechanisms involved in the development of form deprivation myopia and recovery.

During form deprivation in mammals (tree shrews and primates), the sclera weakens due to increased degradation and reduced synthesis of extracellular matrices (ECM) and collagens, and an increased synthesis of matrix metalloproteinase and gelatinase A in the sclera [20,21]. These biologic changes are more prominent at the posterior sclera and are accompanied by axial elongation of the vitreous chamber [10,20-22], indicating that axial elongation is due to the weakening of the sclera in mechanical strength. However, whether the biologic factors found in these studies represent the entire profile of proteins involved in form deprivation myopia is yet to be confirmed.

Two-dimensional gel electrophoresis (2-DE) may provide a guide for selection of more targeted agents in the treatment of functional or pathological disorders and therefore increases the effectiveness of pharmacological manipulation. Results from human 2-DE show that expression of proteins in types and levels is very similar for both the anterior and posterior sclera [23], indicating that functional activities are similar at different locations of the sclera and this ensures a symmetric/or similar morphology at different sites of the sclera. It has been shown by 2-DE that collagen- regulated proteins and 78 kDa glucose-regulated protein (GRP 78, a member of the heat shock protein 70 family) are down-regulated in the sclera of tree shrew eyes developing minus lens-induced myopia [24]. Similar 2-DE results are also found in the retina of mouse eyes developing form deprivation myopia [25]. However, the functional role of these proteins during development of the experimental myopia is still unclear.

Guinea pigs have been increasingly used as an alternative to other species in the study of myopic development and recovery as biometric changes of the guinea pig eye is similar to chickens, tree shrews and monkeys under similar experimental conditions [26-28]. However, changes in protein profiles of various ocular components during myopic development and recovery have not been studied in guinea pigs. This study used 2-DE to investigate protein profiles in guinea pig eyes during form deprivation and recovery. Differential expression levels and types of proteins in posterior sclera of the experimental eye were analyzed between the experimental and normal control eyes.

## Methods

### Experimental design

Forty-eight pigmented guinea pigs (3 weeks old) were randomly assigned to 3 groups (n=16 each group): MD (monocular deprivation for 7 weeks), recovery (re-exposure to normal visual environment after 7-week monocular deprivation) and normal control (free of form deprivation). A facemask was used to induce form deprivation myopia [29,30]. The form deprived eyes in the MD group were used for 2-DE (n=10) and RT–PCR (n=6) after 7 weeks of form deprivation. All right eyes in the normal control group were assigned to 2-DE assessment (n=0) and RT–PCR (n=6) at the time point matching that in the MD group. In the recovery group, the deprived eyes were assessed with 2-DE (n=10) and RT–PCR (n=6) at 4 days after the eyes were re-exposed to normal visual environment. Animals underwent measurements of refraction and vitreous length in all groups before the experiment, at 7 weeks of the experiment in the MD, normal control and recovery groups, and at 4 days after removal of the facemask in the recovery group. All procedures were approved by the institutional Animal Care and Ethics Committee and all procedures complied with the ARVO statement for the use of animals in ophthalmic research.

### Establishment of axial myopia

A latex-made monocularly-deprived facemask (MDF) covered one eye of the animals to induce form deprivation myopia. The procedure of wearing the MDF has been detailed previously [30]. All the animals were raised on a cycle of 12 h illumination (500 Lux) and 12 h darkness daily during the experimental period.

### Biometric measurements

Biometric measurements included streak retinoscopy, keratometry, ultrasonography, and optical coherence tomography in that order. These measurements were performed by a research optometrist with help from an animal care assistant during the cycle of illumination (day time). The optometrist was masked with regard to the identity of treatment in each group. No general anesthesia was necessary for any of the measurement procedures since the animals were very cooperative.

### Retinoscopy

One drop of 1% cyclopentolate hydrochloride (Alcon, Puurs, Belgium) was topically administered to the eye every 5 min for 4 times to achieve a completely dilated pupil. Retinoscopy for all animals was performed by the same optometrist (accuracy: 0.25 D) in a dark room using a streak retinoscope and trial lenses. The refraction was recorded as the mean value of the horizontal and vertical meridians [5,30,31] based on 3 repeated measurements.

### Keratometry

Corneal curvature was measured with a keratometer (OM-4; Topcon, Tokyo, Japan). An 8.00 D lens was attached onto the anterior surface of the keratometer during the measurement to magnify the cornea of the guinea pigs. This allowed readings to be obtained from the steep cornea of the guinea pigs. A group of stainless steel balls with diameters from 5.5 to 11.0 mm were measured by the modified keratometer. Three readings were recorded for each measurement to provide a mean result. The corneal radius of curvature in guinea pigs was then deduced from the readings on the balls with known radii by linear extrapolation [30,32].

### Ultrasonography

A-scan ultrasonagraph (Cinescan A/B; Optikon 2000; S.P.A, Rome, Italy) was used to measure the lens thickness and the vitreous chamber length. The ultrasound frequency was 11 MHz [30,33]. The conducting velocity was assumed to be 1,723.3 m/s for measurement of the lens and 1,540 m/s for measurement of vitreous chambers [30,32,33]. Topical anesthesia was administered with 0.5% proparacaine hydrochloride (Alcon) before the ultrasound measurement. The ultrasound probe had direct contact with the cornea during the axial measurement [34,35]. The tip of the probe had a red light that facilitated the placement of the probe to the corneal apex while the probe was perpendicular to the corneal surface. This perpendicular axis was confirmed by a series of consistent ultrasound traces when realigned on the same eye for repeated measurements. A genuine measurement was confirmed when clear traces of various components of the eye with consistent waves and amplitudes were detected [30,33]. Each of the presented ultrasound data represented averages from 10 repeated measurements.

### Two-dimensional gel electrophoresis and image analysis

The animals were euthanized with an intramuscular injection of an overdose of pentobarbitone sodium. The eyeballs were enucleated with the sclera separated from the other tissues immediately after removal of the cornea. The sclera was then trimmed taking the posterior section which started 16 mm from the limbus and then stored in liquid nitrogen at −196 °C. Prior to 2-DE, the scleral tissue taken from the storage was placed into 200 μl of solution containing 7 M urea, 2 M sulfourea, 4% CHAPS and 1 mM PMSF. The solution was stirred with a glass stirrer, set still for 5 min to achieve a complete dissolution of the cells, followed by centrifugation at 4 °C, 12,000× g for 20 min. The supernatant (soluble proteins) of the solution was extracted and quantified by a modified Bradford assay. The proteins extracted from the posterior sclera of each group were run on three parallel gels (triplicate gels). An equal amount of protein from each sample was loaded onto each gel. Each sample was diluted to a total volume of 350 μl with a buffer containing 8 M urea, 2% CHAPS, 20 mM 1,6-dithiothreitol (DTT), 0.5% immobilized pH gradient (IPG) buffer, and bromophenol blue. Each diluted sample (125 μl) was loaded on the cathode of an IPG strip (13 cm long, pH 3–10, linear; Amersham Pharmacia Biotech, Uppsala, Sweden). The rehydrated strip underwent isoelectric focusing at 20 °C for 11 kVh with a gradually increasing voltage.

After isoelectric focusing, the IPG strips were equilibrated for 15 min twice with a solution of 50 mM Tris-HCL (pH=6.8), 6M urea, 30% glycerol, 2% sodium dodecylsulfate (SDS), and 10 mg/ml dithiothreitol (DDT) with the DTT replaced by 25 mg/ml iodoacetamide (IAM) in the second equilibration. The IPG strips were subsequently transferred onto a vertical slab of 12% SDS-polyacrylamide gels (8.3 cm×7.3 cm). The sample on each gel was run in the other dimension (based on the molecular weight of the proteins) for 0.5 h at 2.5 W and then at 15 W until the dye front reached the gel bottom (Mini-PROTERN3 system; Bio-Rad, Hercules, CA). Each gel was finally stained with Commassie R-250. The image averaged from 3 gels for each sample was analyzed by software (PD Quest 2-D Software; Bio-Rad) to detect differences in expression levels and/or distribution of the protein spots. A protein spot was confirmed only when this spot was detected at the same position on at least two of the triplicate gels, at the same time the image pixel value was converted to the actual optical density, and the average optical density of the protein spot was acted as the synthetic spot. Spots were considered to have different expression levels only when the spot had at least a threefold difference in intensity on at least two of the triplicate gels run by the same sample.

### Trypsin digestion

The protein spots that differed in intensity and location among the MD, recovery, and normal control posterior sclera were excised from the gels, washed with 50 μl Milli-Q water twice (5 min each), and destained with 50 μl of acetonitrile (ACN)/50 mM NH_4_HCO_3_ (1:1, vol/vol) twice for 30 min each. The samples were dehydrated with 50 μl ACN for 20 min, dried, and reduced with a solution of 25 mM NH_4_HCO_3_ containing 10 mM DTT at 56 °C for 1 h. The samples were then alkylated by 25 mM NH_4_HCO_3_ containing 55 mM iodoacetamide in darkness at room temperature for 45 min and washed with 25 mM NH_4_HCO_3_, 50% acetonitrile, and 100% acetonitrile for 10 min, respectively. Enzymatic digestion was performed by adding the samples to 0.1 mg/ml trypsin at a final ratio of substrate to trypsin of 40:1 wt/wt and incubation at 37 °C for 12 h, followed by termination with 2.5% trifluoroacetic acid (TFA).

### Matrix-Assisted Laser Desorption Ionization-Time of Flight Mass Spectrometry (MALDI-TOF)

Samples digested by trypsin (the differential protein spots in posterior sclera among the MD, recovery and normal control eyes) were mixed with *a*-cyano-4-hydroxycinnamic acid (2:1, vol/vol) in an Eppendorf tube. Two microliters of the mixtures were added to a goldplated sample holder, dried and transferred into the MALDI-TOF mass spectrometer (Autoflex; Bruker Daltonics, Billerica, MA). The monoisotopic peptide masses were searched based on human cDNA and protein databases in NCBI (Mascot; Matrix Science Ltd, London, UK) with a mass accuracy at 50 ppm for the parent ion mass. The proteins were identified functionally based on (1) the Molecular Weight Search (MOWSE) score above the 5% significance threshold from the database (Molecular Weight Search, Human Genome Mapping Project Resource Centre, Sanger Centre), (2) the theoretical p*I* and the molecular weight (MW) of the search result matching the 2-DE position of the corresponding spot, and (3) less than 20% uncleaved peptides in the matching sequence.

### Reverse Transcriptase Polymerase Chain Reaction (RT–PCR)

RT–PCR was run to detect whether changes in levels of crystallins (Table 1) were regulated by changes in mRNA expression of the associated genes. Total RNA was extracted from the posterior sclera with Trizol reagent (Invitrogen, Grand Island, NY) and confirmed using spectrophotometry and formaldehyde/agarose gel electrophoresis. To remove contaminating genomic DNA, 1 μg of total RNA was treated with 1 U RNase free DNase I (Promega, Madison, WI) at 37 °C for 30 min and then heated with 1 μl stop solution (Promega) at 65 °C for 10 min. Subsequently, 0.5 μg of total RNA in each sample was reversely transcribed (M-MLV reverse transcriptase, Promega) using 0.04 μg random primers (Promega) in a total volume of 20 μl according to manufacturer’s instructions.

**Table 1:** Sequence of primer pairs and length of the amplified sequences based on the NCBI database

**Gene**	**Forward primer (5′-3′)**	**Reverse primer (5′-3′)**	**Annealing temperature (°C)**	**Cycles**	**Length of product (bp)**
αA-crystallin	AGCCCTTGCCAGCCATCT	GCTTGTGCCACCTGCTCTTTA	60	33	220
αB-crystallin	AGTTCTTCGGAGAGCACCTGTT	TCCTTGGTCCATTCACAGTGAG	64	34	321
βA3/A1-crystallin	GCCTGGAGTGGAAGCAAT	CTGGATACGGCGAATAGA	59	35	344
βA4-crystallin	TGCTGAGTGGAGCGTGGGTAGG	GTGGACGTGGAAGGAGCCCACT	65	35	300
βB2-crystallin	CCAGAACCTTAACCCCAAGATC	GCTGTCCACTTTGATGGGCCTC	65	35	277
GAPDH	CGGAGTCAACGGATTTGGTCGTAT	AGCCTTCTCCATGGTGGTGAAGAC	65	30	304

Due to the lack of information on crystallin gene sequences for guinea pigs, primers were designed using mouse αA-crystallin, αB-crystallin, βA3/A1-crystallin, βA4-crystallin, and βB2-crystallin sequences in areas of high interspecies identity (Table 1). Taq polymerase (Ex Taq; Takara, Dalian, China) and 1 μM primers were used for PCR amplification. The amplification process included: pre-denaturation for 5 min at 95 °C, followed by 30–35 °C cycles of denaturation at 95 °C for 30 s, annealing at 59–65 °C for 30 s, extension at 72 °C for 40 s, and a final extension at 72 °C for 10 min. The exact number in cycles and annealing temperature were optimized based on different genes tested (Table 1). Equal volume (5µl) of all PCR products was electrophorezed on 2% agarose gels containing 2 μl goldview (SBS genetech, Beijing, China) and then photographed under ultraviolet illumination. The sizes of the amplified products were estimated using Marker DL2000 (Takara).

### Statistical analysis

The refractive status and axial components of the form-deprived eyes were statistically compared to those of the fellow eyes within the same group at each time point (paired sample *t*-test, SPSS Version 11.5; SPSS Inc., 1989-1999, Chicago, IL). These biometric results were also compared between different groups (one-way ANOVA with Bonferroni correction, SPSS Version 11.5). Both the intra-group and inter-group differences were determined as significant at p<0.05 and highly significant at p<0.01.

## Results

### Confirmation of phenotypic changes induced by form deprivation

Refraction of the guinea pig eyes in all groups was hyperopic before the experiment (3 weeks of age) with no significant differences between eyes of the same animals in refraction and vitreous length (p values were 0.21 and 0.95, respectively, paired sample *t*-test, Table 2). The MD eyes developed myopia of −4.24 D with a greater vitreous length (0.22 mm) when compared to the fellow eyes over the period of 7 weeks. In the recovery group, the MD eyes (−3.69 relative to the fellow eye) developed toward hyperopia after removal of the facemask with a relative myopia of −2.85 at 4 days of the recovery process. There was no significant difference in changes of refraction and axial components between the fellow eyes of either the MD group or the recovery group and the normal control eyes.

**Table 2:** The refractive state and vitreous length in different groups.

** **	**Refraction (D)**					**Vitreous length (mm)**				
** **	** **	**MD group**		**Recovery group**		** **	**MD group**		**Recovery group**	
**Age**	**NC group**	**MD eyes**	**Fellow eyes**	**Recovery eyes**	**Fellow eyes**	**NC group**	**MD eyes**	**Fellow eyes**	**Recovery eyes**	**Fellow eyes**
3-w	4.92±0.69	4.46±0.27	3.92±0.31	4.11±0.28	3.57±0.44	3.37±0.02	3.42±0.02	3.42±0.03	3.45±0.04	3.43±0.05
10-w	4.10±0.72	−0.74±0.51^a,b^	3.50±0.34	−0.60±0.44^a,b^	3.09±0.49	3.47±0.08	3.76±0.02^a,b^	3.54±0.03	3.78±0.02^a,b^	3.50±0.03
10-w+4d	-	-	-	1.46±0.43^c^	4.31±0.48	-	-	-	3.76±0.02^d^	3.54±0.04

NC group: Normal Control group; MD group: Monocular deprivation group; ^a^p<0.01 form-deprived eyes versus fellow eyes at the end of the form deprivation (10 weeks old); ^b^p<0.05 the paired *t*-test before and after form deprivation in the experiment eyes; ^c^p<0.01 the paired *t*-test before and after the removing of the facemask in the recovery eyes; ^d^p=0.48 the paired *t*-test before and after the removing of the facemask in the recovery eyes.

### Protein profiles of the posterior sclera on 2-DE

**Figure 1:**
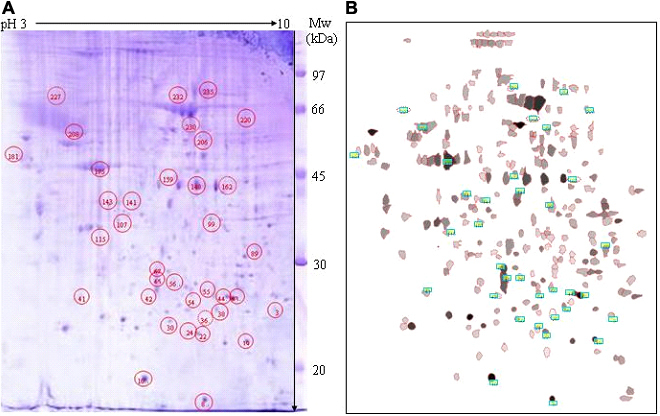
Protein profiles of the posterior sclera on 2-DE. The 2-DE distributing profile of the differentially expressed protein spots from the posterior sclera distinct between the normal control eyes and the MD eyes or the recovery eyes (**A**). Synthetic gel produced from triplicate gels of normal control eyes (**B**), the differentially expressed protein spots were framing labeled.

**Figure 2:**
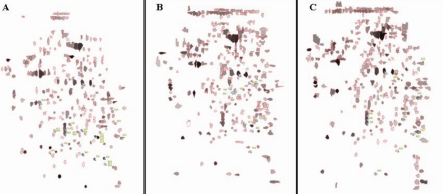
Dynamic 2-DE profiles of the differentially expressed proteins in the posterior sclera of different groups. **A**: normal control eyes (NC), **B**: monocular deprivation eyes (MD), **C**: recovery eyes.

The 2-DE profile of all protein spots from the posterior sclera was distinct between the MD and recovery or the control eyes (Figure 1 and Figure 2). Twenty-six spots were different between the MD eyes and normal control eyes in either the level or location of expression on 2-DE (Table 3). These included 18 spots with a different level of expression between the 2 groups where the MD eyes had 3 spots (2 identified: 36, 45) only detected in the MD eyes, 5 spots (4 identified: 3, 42, 181, 232) with a higher expression in the MD eyes, 13 spots (8 identified: 22, 67, 89, 143, 159, 206, 208, 235) with a lower expression in the MD eyes, and 5 spots (4 identified: 24, 41, 44, 99) detected only in the normal control eyes (Table 3).

**Table 3:** Protein expression in posterior sclera: MD eyes versus normal control eyes (NC).

**Spot no.^a^**	**Protein level**	**Fold**	**Accession number^b^**	**Protein description**	**Protein score^c^**
3	Up	3.2	gi|61365690	adenylate kinase 1	119
42	Up	8.8	gi|73995384	beta A4-crystallin	101
181	Up	3.5	gi|78195022	Fe-S type hydro-lyases tartrate/fumarate alpha region	66
232	Up	10.5	gi|68417708	Ca^2+^-dependent activator protein for secretion 2 isoform b, partial	41
36	Up	N/MD^d^	gi|61822969	junctophilin 1, partial	42
45	Up	N/MD	gi|13936373	glutathione S-transferase subunit gYc	132
22	Down	3.5	gi|4557920	G12v mutant of human placental Cdc42 GTPase in the GDP form chain B	123
67	Down	3.6	gi|12407849	peroxiredoxin 4	94
89	Down	3.4	gi|32400726	putative alpha-tubulin	97
143	Down	3.6	gi|60813640	eukaryotic translation initiation factor 3 subunit 2 beta	82
159	Down	3.0	gi|73980918	macrophage capping protein (Actin-regulatory protein CAP-G)	103
206	Down	3.3	gi|25777724	aldehyde dehydrogenase 1A2 isoform 1	91
208	Down	3.2	gi|76618159	tubulin alpha-2 chain	126
235	Down	3.3	gi|78167676	ATPase	52
24	Down	^e^D/NC^m^	gi|1001946	adenine phosphoribosyltransferase	95
41	Down	D/NC^m^	gi|55824562	peroxiredoxin 1	179
44	Down	D/NC^m^	gi|6978713	beta B2-crystallin	148
99	Down	D/NC^m^	gi|52138659	guanine nucleotide binding protein (G protein), beta polypeptide 2-like 1	221

^a^Spot no. is the unique sample spot protein number assigned by the PDQuest software. ^b^Accession number is the MASCOT result of MALDI-TOF searched from the NCBInr database. ^c^Protein score was from MALDI-TOF identification. The proteins that had a statistically significant protein score of great than 76 (p<0.05) were considered successfully identified. ^d^N/MD, the protein spots were newly induced in posterior sclera of the MD eyes. ^e^D/NC^m^, the protein spots were only detected in posterior sclera of the normal control eyes.

A comparison between the recovery and normal control eyes showed 3 spots (2 identified: 230, 232) with a higher expression, 19 spots (7 identified: 10, 19, 30, 38, 43, 141, 166) with a lower expression in the recovery eyes, 6 spots (5 identified: 44, 54, 55, 56, 115) only found in the normal control eyes and 5 (2 identified: 8, 162) only found in the recovery eyes (Table 4). The MD eyes (in the MD group) had 5 spots with 2 (all identified: 107, 227) having a lower expression and 3 (all identified: 148, 175, 220) having a higher expression compared to the recovery eyes (Table 5).

**Table 4:** Protein expression in posterior sclera: recovery eyes versus normal control eyes (NC).

**Spot no.^a^**	**Protein level**	**Fold**	**Accession number^b^**	**Protein description**	**Protein score^c^**
220	Up	3.0	gi|21614520	glucose-6-phosphate dehydrogenase	90
232	Up	10.5	gi|68417708	Ca^2+^-dependent activator protein for secretion 2 isoform b, partial	41
8	Up	^d^N/R	gi|11968098	ADP-ribosylation factor 1	115
162	Up	N/R	gi|50513041	phosphoglycerate kinase 1	81
10	Down	4.7	gi|117360	alpha A-crystallin	147
19	Down	3.6	gi|265053	alpha B-crystallin	175
30	Down	3.9	gi|73995384	beta A4-crystallin	107
38	Down	3.5	gi|14285262	beta A3/A1-crystallin	154
43	Down	5.1	gi|299263	beta B2-crystallin	269
141	Down	3.2	gi|73996530	tubulin alpha-6	131
166	Down	3.2	gi|1703112	actin cytoskeletal 2 (LPC2)	81
44	Down	^e^D/NC^r^	gi|6978713	beta B2 crystallin	148
54	Down	D/NC^r^	gi|50979116	heat-shock protein	79
55	Down	D/NC^r^	gi|27311829	putative DEAD/DEAH box helicase	61
56	Down	D/NC^r^	gi|14285262	beta A3/A1-crystallin	103
115	Down	D/NC^r^	gi|1006831	ANXA5 protein	101

^a^Spot no. is the unique sample spot protein number assigned by the PDQuest software. ^b^Accession number is the MASCOT result of MALDI-TOF searched from the NCBInr database. ^c^Protein score was from MALDI-TOF identification. The proteins that had a statistically significant protein score of great than 76 (p<0.05) were considered successfully identified. ^d^N/R, the protein spots were newly induced in posterior sclera of the recovery eyes. ^e^D/NC^r^, the protein spots were only detected in posterior sclera of the normal control eyes.

**Table 5:** Protein expression in posterior sclera: MD eyes versus recovery eyes.

**Spot no.^a^**	**Protein level**	**Fold**	**Accession number^b^**	**Protein description**	**Protein Score^c^**
107	Up	3	gi|68270947	capping protein (actin filament) muscle Z-line alpha 2	101
227	Up	3.7	gi|73957579	similar to T-complex protein 1, zeta subunit (TCP-1-zeta) (CCT-zeta) (CCT-zeta-1) isoform 3	95
148	Down	3.5	gi|54016803	putative amidase [Nocardia farcinica IFM 10152]	72
175	Down	9.5	gi|73964667	hypothetical protein XP_533132	210
220	Down	3.5	gi|21614520	glucose-6-phosphate dehydrogenase	90

^a^Spot no. is the unique sample spot protein number assigned by the PDQuest software. ^b^Accession number is the MASCOT result of MALDI-TOF searched from the NCBInr database. ^c^Protein score was from MALDI-TOF identification. The proteins that had a statistically significant protein score of great than 76 (p<0.05) were considered successfully identified.

### RT–PCR results

**Figure 3:**
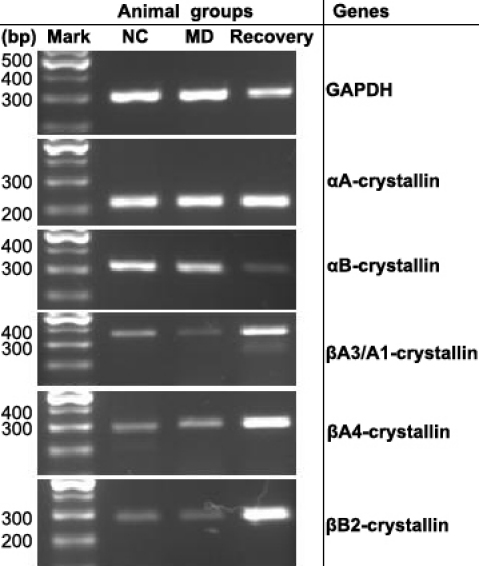
Expression of αA-, αB-, βA3/A1-, βA4- and βB2-crystallins mRNA in posterior sclera of the guinea pigs in different groups. normal control eyes (NC), monocular deprivation eyes (MD), recovery eyes. *GAPDH* was used as a loading control.

In the normal control eyes, the level of crystallin mRNA expression was highest for αA-crystallin and αB-crystallin, followed by βA3/A1-, βA4- and βB2-crystallins (Figure 3). The expression of αA-crystallin mRNA did not change in the MD eyes, but was higher in the recovery eyes when compared to the normal control eyes. The expression of αB-crystallin mRNA was lower in both the MD and recovery eyes compared to the normal control eyes, and such a decrease in the recovery eyes was more obvious than in the MD eyes. The expression of βA3/A1-crystallin mRNA decreased in the MD eyes but obviously increased in the recovery eyes when compared to the normal control eyes. The expression of βA4-crystallin mRNA in both the MD and recovery eyes increased compared to the normal control eyes and such an increase in the recovery eyes was more obvious than in the MD eyes. The expression of βB2-crystallin mRNA decreased in the MD eyes but increased in the recovery eyes when compared to the normal control eyes.

## Discussion

In this study, guinea pig eyes treated with monocular deprivation facemask (MDF) for a period of 7 weeks developed myopia with a degree of at least −4.24 D and had a greater increase in vitreous length by 0.22 mm (p<0.001) when compared to the fellow eyes (Table 2). This axial myopia shifted rapidly toward hyperopia with a slowing of the growth in the vitreous length within 4 days of removal of the MDF. These results are similar to those found in previous studies on the same species [29,30] and confirm that the eyes used were indeed myopic.

Most of the proteins found to have different levels of expression have not been studied for their roles in the development of various axial components of the eye. Presumably, any proteins that were upregulated in the MD eyes (compared to normal control eyes) were needed to facilitate the remodeling of the sclera, and those that were down-regulated needed to be dampened to allow scleral remodeling. Both junctophilin 1 (JP1), a triadic protein that is thought to be required for normal voltage-gated sarcoplasmic reticulum Ca^2+^ release [36] and GST subunit gYc with glutathione transferase activity were only detected in the MD eyes and so were probably very highly upregulated during MD. Four proteins that were also upregulated in the MD eyes included adenylate kinase 1 (AK1) that catalyzes conversion between ATP and AMP molecules and presumably indicates a need for more energy during scleral remodelling, βA4-crystallin which seems to be essential for ocular growth, as defects in the gene encoding this protein are a cause of microphthalmia [37], Fe-S hydrolyase which is also involved in energy production (possibly from NADH), and Ca^2+^-dependent activator protein for secretion 2 isoform b (CAPS2b) which is a secretory vesicle-associated protein involved in the release of neurotrophin.

Of the four proteins that were undetected in the MD eyes as compared to the normal control eyes, and thus heavily down-regulated during formation of MD, adenine phosphoribosyltransferase (APRT) is an intracellular enzyme that contributes to formation of AMP by catalyzing the phosphoribosylation of adenine, peroxiredoxin 1 acts to protect cells from oxidation, βB2-crystallin is involved in post-translational modification of proteins, and Gnb2l1 (guanine nucleotide binding protein [G protein], beta polypeptide 2-like 1) mediates signal transduction. Another eight proteins were down-regulated in the MD eyes: G12v mutant of human placental Cdc42 GTPase in the GDP form chain B which is a GTP-binding protein, which involves in actin assembly, peroxiredoxin 4 which is an anti-oxidative enzyme involved in gene transcription [38], eukaryotic translation initiation factor 3 subunit 2β (eIF3–2β) which promotes protein translation from tRNAi and mRNA, macrophage capping protein which is an actin-regulatory protein, aldehyde dehydrogenase 1 A2 isoform 1 (ALDH1A2) that catalyzes the synthesis of retinoic acid in the retina, tubulin α-2 (a cytoskeleton constituent protein) and ATPase that catalyzes the decomposition of ATP (ATP) into adenosine diphosphate (ADP) and free phosphate.

Two proteins were only expressed in the recovery eyes: ADP-ribosylation factor 1 (ARF1), a small GTP-binding protein of the Ras superfamily, which functions as a regulator of vesicular traffic and actin remodeling and phosphoglycerate kinase 1 (PGK1) which promotes reduction of plasmin disulfide bonds leading to angiostatin formation and inhibition of tumor angiogenesis. Five spot proteins that were undetected in the recovery eyes and so presumably heavily down-regulated during the recovery process included the heat-shock protein which involves in cell survival under hyperthermia and other environmental stresses, βB2-crystallin, the putative DEAD/DEAH box helicase which is needed to unwind nucleic acids, βA3/A1-crystallin, and ANXA5 protein which is involved in apoptosis [39]. Seven other proteins were down-regulated in the recovery eyes, including αA-crystallin, αB-crystallin, βA4-crystallin, βA3/A1-crystallin (in a different position on the gel than the spot identified above), βB2-crystallin (in a different position on the gel than the spot identified above), tubulin α-6 and actin cytoskeletal 2 (LPC2). Down-regulation of tubulins (tubulin α-2 and tubulin α-6) and CAP-G (macrophage capping protein) may result in a decreased movement of cells and internal organelles during scleral remodelling.

A comparison between the MD and the recovery eyes showed that 2 proteins were upregulated in the recovery eyes: CAPZA2 [capping protein (actin filament) muscle Z-line alpha 2, which regulates the growth of the actin filaments at the barbed end], and CCT6A3 (T-complex protein 1, zeta subunit [TCP-1-zeta; CCT-zeta; CCT-zeta-1] isoform 3) which is a member of the chaperonin containing TCP1 complex (CCT); whereas a putative amidase (an enzyme that hydrolyzes amide), glucose-6-phosphate dehydrogenase (G6PD, a cytosolic enzyme in the pentose phosphate pathway that supplies reducing energy to cells by maintaining the level of the co-enzyme nicotinamide adenine dinucleotide phosphate [NADPH]), and a hypothetical protein with the spot matched as beta and gamma cytoplasmic actin were down-regulated. These differentially expressed proteins belong to the following functional categories: cytoskeleton, metabolic, redox, protein degradation, apoptosis and heat shock protein/chaperones and these categories suggest that the altered proteins appear to have multiple roles in scleral remodeling, increase of extracellular matrix (ECM) and changes in scleral collagen fibers during the development of and recovery from form deprivation myopia.

Crystallins are major structural proteins in the lens and belong to the small heat-shock protein family which control protein folding in the endoplasmic reticulum and subsequent intracellular trafficking [40]. In spite of their diversity, many crystallins play a role in essential developmental processes such as cell elongation and inhibition of cell apoptosis [41]. In the retina, the expression of members of α-, β- and γ-crystallin gene families are upregulated under stress, induced by intense light exposure or retinal tearing [42-44]. However, the precise organization and biologic functions of the crystallins in the sclera are not known. In this study, crystallins were located between p*I* 3–10 which is similar to those from a previous 2-DE study of chickens [45]. Crystallins including subtypes of αA-, αB-, βA3/A1-, βA4- and βB2-crystalin were detected at both mRNA (RT–PCR) and functional levels (2-DE) in the posterior sclera of the guinea pigs after form deprivation and the recovery.

For the α-crystallins, two α-crystallin polypeptides (αA and αB) are molecular chaperones that can protect proteins (e.g., β-crystallins) from thermal aggregation [46-49], with αB-crystallin being more efficient than αA-crystallin in preventing the aggregation of proteins [48,49]. The αA and αB subunits share approximately 60% amino acid sequence identity and account for 20% to 30% of the lens total proteins [50]. They exist as heteromers that can undergo subunit exchange [51]. The α-crystallins may be involved in the regulation of cellular growth and genomic stability [52-54]. It has been found that αA-, βA3/A1-, βB1-, and βB2-crystallin mRNAs are upregulated in retina-RPE-choroid of form-deprived chicken eyes [55]. Retinal expression of αB-crystallin mRNA elevates in form-deprived eyes and remains elevated with only a slight return to control levels after the re-exposure of the eye to normal visual conditions in chickens [56,57]. These results indicate that members of the crystallin family do play a role in the development of form deprivation myopia.

In this present study, the expression of αB-crystallin in the posterior sclera was down-regulated in the myopic and recovery eyes at both protein and mRNA levels compared to the normal control eyes (Table 6). This is different to previous studies [56,57] where αB-crystallin mRNA was upregulated in retina of the myopic and recovery eyes in chickens. This may be due to the use of different species or different tissues for the recovery of the mRNA. The level of αA-crystallin mRNA or protein expression did not obviously differ in the myopic eyes compared to the normal control eyes. Furthermore, αA-crystallin protein in the recovery eyes was down-regulated although the αA-crystallin mRNA is upregulated. A similar mismatch in expression of βA3/A1-, βA4- and βB2-crystallins between 2-DE and PCR occurred in the recovery eyes. It is possible that these mRNA changes are a prelude to protein changes that only manifest, or at least can be detected to change, after 4 days of recovery.

**Table 6:** Expression of αA-, αB-, βA3/A1-, βA4- and βB2-crystallins in posterior sclera of the guinea pigs in monocular deprivation eyes (MD), recovery eyes comparing to normal control eyes.

**Groups**		**αA**	**αB**	**βA3/A1**	**βA4**	**βB2**
Protein levels	MD	-	-	-	↑	↓
	Recovery	↓	↓	↓	↓	↓
mRNA levels	MD	-	↓	↓	↑	↓
	Recovery	↑	↓	↑*	↑*	↑*

“↑”: upregulation, “↓”: down-regulation, “-”: difference less than threefold, “*”: significant difference.

This present study focused on changes in protein profiles of posterior sclera of guinea pigs, whereas Frost and Norton [24] examined the entire sclera of tree shrews. An inherent variability across the gels might have limited the detection of biologic variations between groups, although Diz [58] has found that pooled samples matched the mean expression of the individuals making up the pool for the majority of proteins. Hence, results in the present study are likely to provide a good survey of the number of proteins whose levels change during form deprivation myopia and the subsequent recovery. Previous studies have demonstrated changes to other extracellular matrix proteins including collagen I and glucosaminoglycan [17,24]. However, the present study did not identify changes to these proteins using 2-DE. This may have been due to the cut-off of threefold in protein expression before spots on the 2-DE were identified, and changes to these proteins not reaching this cut off.

In summary, this study adopted a gel-based proteomic approach to probe the serially changed proteins in posterior sclera of guinea pigs during development of form deprivation myopia and recovery. In general, changes in crystallin protein levels were consistent with those in mRNA levels during the development of FDM. However, changes in most crystallin protein levels were mismatched with their mRNA levels during the recovery stage. Furthermore, the absolute quantities of mRNA expression for the proteins isolated by 2-DE may not be accurate without real time PCR. However, the main interest for this study was to investigate the difference in levels of protein expression between different experimental groups rather than the absolute quantity for the individual proteins. Further proteomic analyses will be focused on the functional role of the differentially expressed proteins during development of the experimental myopia and recovery.
